# Dual-ligand modified liposomes provide effective local targeted delivery of lung-cancer drug by antibody and tumor lineage-homing cell-penetrating peptide

**DOI:** 10.1080/10717544.2018.1425777

**Published:** 2018-01-15

**Authors:** Congcong Lin, Xue Zhang, Hubiao Chen, Zhaoxiang Bian, Ge Zhang, Muhammad Kashif Riaz, Deependra Tyagi, Ge Lin, Yanbo Zhang, Jinjin Wang, Aiping Lu, Zhijun Yang

**Affiliations:** a School of Chinese Medicine, Hong Kong Baptist University, Hong Kong, China;; b School of Biomedical Sciences, Chinese University of Hong Kong, Hong Kong, China;; c School of Chinese Medicine, Li Ka Shing Faculty of Medicine, The University of Hong Kong, Hong Kong, China;; d Changshu Research Institute, Hong Kong Baptist University, Changshu Economic and Technological Development (CETD) Zone, Changshu, China

**Keywords:** Dual-ligand liposomes, carbonic anhydrase IX, tumor lineage-homing cell penetrating peptide, pulmonary delivery, orthotopic lung cancer model

## Abstract

The abilities of a drug delivery system to target and penetrate tumor masses are key factors in determining the system’s chemotherapeutic efficacy. Here, we explored the utility of an anti-carbonic anhydrase IX (anti-CA IX) antibody and CPP33 dual-ligand modified triptolide-loaded liposomes (dl-TPL-lip) to simultaneously enhance the tumor-specific targeting and increase tumor cell penetration of TPL. *In vitro*, the dl-TPL-lip increased the cytotoxicity of TPL in CA IX-positive lung cancer cells, which showed tunable size (137.6 ± 0.8 nm), high-encapsulation efficiency (86.3 ± 2.6%) and sustained release. Dl-TPL-lip significantly improved the ability of liposomes to penetrate 3 D tumor spheroids and exhibited a superior inhibiting effect. Furthermore, pharmacokinetic studies in rats that received TPL liposomal formulations by endotracheal administration showed a reduced concentration of TPL (17.3%–30.6% compared to free TPL) in systemic circulation. After pulmonary administration in orthotopic lung tumor-bearing mice, dl-TPL-lip significantly enhanced TPL anti-cancer efficacy without apparent systemic toxicity. This dual-ligand modified liposomal vehicle presents a potential system for localized and targeted delivery of anti-cancer drugs to improve their efficacy.

## Introduction

To be effective, chemotherapeutics must selectively reach the specific tumor site and then penetrate cells in a sufficient concentration to kill the cancer cells without harming neighboring healthy cells (Yao et al., 2016). In other words, site-specific delivery, efficient penetration of the anti-cancer drugs into tumors, and lack of side effects are crucial factors for the success of cancer chemotherapy (Minchinton and Tannock, 2006). Conventional liposomes have achieved two of these goals, namely, enhanced tumor delivery and decreased side effects; however, they accumulate passively in the tumor area rather than actively penetrating cancer cells (Maeda et al., 2013).

Recently, active targeted liposomes modified with antibodies or other ligands have attracted researchers’ interest. These liposomes display improved anti-cancer therapeutic efficacy because they bind specifically with the over-expressed receptors of tumor cells (Al-Ahmady et al., 2014; Dicheva et al., 2014; She et al., 2014). However, they are not being used in clinical applications because (a) binding-site barriers prevent them from penetrating deeply into tumors, and (b) the dynamic and heterogeneous nature of tumor cells prevents them from efficient binding (Cai et al., 2014b). Therefore, the development of a dual-ligand delivery platform to insure both targeted accumulation and penetration is now being explored as a potentially more effective strategy for fighting cancer.

The poor vascular organization and extra consumption of oxygen by solid tumors lead to low-oxygen tension in the tumor microenvironment (Raghunand et al., 2003). The core cellular response to hypoxia is the activation of hypoxia-inducible factor (HIF), which results in the expression of carbonic anhydrase IX (CA IX) on the tumor cell surface (Pastorek and Pastorekova, 2015). CA IX is reported aberrantly expressed in various tumors including the lung (Vermylen et al., 1999; Swinson et al., 2003; Le et al., 2006), kidney (Liao et al., 1997), colon (Saarnio et al., 1998), and breast (Wykoff et al., 2001), whereas its expression in normal tissue is restricted. Thus, CA IX has been identified as an attractive target for anti-cancer therapy (McDonald et al., 2012). Recently, CA IX-targeted immuno-liposomes have been developed by our group and shown to have improved therapeutic effect in lung cancer mouse models (Lin et al., 2017). Whether the therapeutic index can be further improved by enhancing the penetration of this CA IX-targeting immuno-liposomes has attracted our interest. Furthermore, whether dual-ligand liposomes can be created to target the same cells for lung cancer therapy has not been investigated. This study explores these two possibilities.

The penetration of liposomes into the inner parts of tumor is a key factor for governing efficient delivery of anti-cancer drugs to cancer cells (Bae & Park, 2011). However, the hydrophobic nature of cellular membranes often protects cells from an influx of exogenous molecules, including bioactive molecules such as proteins and oligonucleotides (Wang et al., 2014). CPP33, a novel tumor lineage-homing cell-penetrating peptide, with a sequence of RLWMRWYSPRTRAYG has been synthesized using mRNA display technology (Kondo et al., 2012). This CPP33 has the ability to penetrate the cell membrane of human non-small cell lung cancer (NSCLC) A549 cells, while minimizing the transduction into normal tissue (Kondo et al., 2012). Modification of liposomes with CPP33 was expected to facilitate the delivery of cargo molecules into the NSCLC cells (Fonseca et al., 2009).

Furthermore, the pharmacokinetic advantages, anti-cancer effects and reduced systemic side effects of pulmonary administration for metastatic or primary lung cancer have been demonstrated compared to intravenous administration (Koshkina et al., 2001; Wauthoz et al., 2010; Kaminskas et al., 2014). Pulmonary delivery of anti-cancer drugs could increase exposure of the lung to active ingredients and minimize adverse effects on healthy organs by limiting drug concentration in the blood (Garbuzenko et al., 2010).

Therefore, we designed anti-CA IX antibody and CPP33 dual-ligand liposomes for efficiently delivering triptolide (TPL) to NSCLC by pulmonary administration. In this study, anti-CA IX antibody and CPP33 were conjugated with DSPE-PEG_2000_-maleimide first, and then the liposomal surface were decorated with conjugates to achieve active targeting. The dual-ligand liposomes with anti-CA IX and CPP33 were expected to simultaneously enhance the specific-targeting efficacy via antibody-antigen binding and increase the penetration into lung tumor cells. TPL, an active compound isolated from the Chinese medicinal plant *Tripterygium wilfordii* Hook F, which has a poor solubility in water (0.017 mg/mL) was selected as the anti-cancer drug (Chen et al., 2012a; Reno et al., 2015; Li et al., 2016). The characterization of dual-ligand TPL-liposomes (dl-TPL-lip) including particle size, entrapment efficiency and drug release properties was carried out. The effects of dl-TPL-lip on NSCLC cells were evaluated by wound healing and apoptosis assay. Moreover, the penetrating ability and inhibition efficacy of dl-TPL-lip were further investigated using 3 D tumor spheroids. The pharmacokinetic and pharmacodynamics behaviors of dl-TPL-lip *in vivo* were studied by pulmonary administration.

## Materials and methods

### Materials

The mouse monoclonal anti-CA IX antibody (MN, CA IX, 214274) was purchased from US Biological Life Sciences (Salem, USA). CPP33 peptide with a terminal cysteine (Cys-CPP33) was synthesized by Scilight Biotechnology (Beijing, China). Soybean lecithin (SPC) was purchased from Shanghai Tai Wei Chemical Company (Shanghai, China). Triptolide was supplied by Chengdu Biopurify Phytochemicals Ltd. (Sichuan, China). 1,2-Dipalmitoyl-sn-glycero-3-phosphoethanolamine-N-(7-nitro-2-1,3-benzoxadiazol-4-yl) [triethylamine salt] (NBD-DPPE) and N-[(3-maleimide-1-oxopropyl) aminopropylpolyethylene-glycol-carbamyl] distearoylphosphatidyl-ethanolamine (DSPE-PEG-MAL) were purchased from NOF America Corporation (White Plains, NY, USA).

The luciferase-expressing A549 cells (A549-Red-FLuc) were purchased from PerkinElmer, Inc (Waltham, USA). Male Sprague Dawley rats (250 ± 20 g) were obtained from Laboratory Animal Services Center (HongKong, China), the Chinese University of Hong Kong. Male BALB/c nu/nu mice (7–8 weeks old) were supplied by Tian Hang Technology Limited (Hongkong, China). All animal experiments involved were approved by the Health Department of Hong Kong Special Administrative Region and conducted according to the guidelines of the Committee on the use of Human and Animal Subjects in Teaching and Research of Hong Kong Baptist University.

### Synthesis of DSPE-PEG-MAL-CPP33 conjugate

Briefly, 20 mg of DSPE-PEG_2000_-MAL was dissolved in HEPES buffer (20 mM HEPES, 10 mM EDTA-2Na, pH 6.5), and then cysteine-modified CPP33 (Cys-CPP33, 14.1 mg, 35.6 μmol) dissolved in 20% of acetonitrile was added into the DSPE-PEG_2000_-MAL solution. The reaction was continued for 48 h with gent stirring at room temperature under nitrogen protection. Then, the reaction mixture was incubated with l-cysteine (10 times of the molar ratio to maleimide residues) for another 4 h to cap unreacted maleimide group. The resulting product was dialyzed against distilled water for 48 h (MWCO 3500 Da). At last, the purified conjugate was lyophilized and stored at −20 °C. The conjugation of CPP33 with DSPE-PEG_2000_-MAL was authenticated using a Matrix-assisted laser desorption/ionization-time of flight mass spectrometer (MALDI-TOF MS, Bruker Daltonics, Billerica, Germany).

### Preparation of dl-TPL-lip

CPP33-modified TPL-loaded liposomes (CPP33-TPL-lip) with lipid composition of SPC:DSPE-PEG_2000_:DSPE-PEG_2000_-MAL-CPP33 (97:3:1, mol/mol) and TPL-loaded liposomes (TPL-lip) with lipid composition of SPC:DSPE-PEG_2000_ (97:3, mol/mol), were prepared by ethanol injection method followed by extrusion (Wong et al., 2014). Briefly, all lipid and TPL was dissolved thoroughly with ethanol and subsequently injected into 2 mL phosphate buffered saline (PBS, pH 7.4) under stirring at 60 °C using a syringe needle, and kept stirring for 1 h. Finally, the dispersion was passed through a LIPEX^TM^ Extruder (NorthernLipids, Vancouver, Canada) using polycarbonate filter, first with a pore size of 200 nm three times, then through a filter with 100 nm-sized pores three times, and finally through a filter with 80 nm-size pore five times.

Dl-TPL-lip and CA IX-modified TPL-loaded liposomes (CA IX-TPL-lip) were developed using the post-insertion technique as described previously (Lin et al., 2017). In brief, DSPE-PEG-MAL micelles were prepared by film hydration at the concentration of 6 mM. The anti-CA IX antibody was thiolated at the hinge region by DTT (0.5 mM) at room temperature for 90 min in the presence of EDTA (10 mM) (Mahmoud et al., 2011). Subsequently, the obtained half antibody with a free sulfhydryl group was coupled with DSPE-PEG-MAL micelles at a molar ratio of 1:60 by incubation at 4 °C with gentle agitation overnight. CA IX-conjugated micelles were incubated with preformed CPP33-TPL-lip and TPL-lip at 60 °C for 2 h. The unincorporated liposomes were removed using a Sepharose CL-4B gel column (Sigma-Aldrich, Darmstadt, Germany). The same procedures were followed to prepare NBD-DPPE labeled liposomes, except the TPL was substituted by NBD-DPPE (1% molar ratio).

## Characterization of dl-TPL-lip

Size and polydispersity index (PDI) of liposomes were determined with Delsa Nano HC Particle Analyzer (Beckman Coulter, Brea, CA, USA). The ultrafiltration technique was used to separate the unencapsulated TPL from the liposomal formulations. A total of 250 μL TPL liposomal formulations was placed in the upper chamber of the Amicon Ultra-0.5 centrifugal filter (10 K cut-off) (Millipore Co., Bedford, MA) and was centrifuged at 10,000 rpm for 15 min. The amount of unencapsulated TPL in the ultra-filtrate was determined with ultra-performance liquid chromatography (UPLC, ACQUITY UPLC System, Waters, Milford, MA, USA) with UV detection at 230 nm. The mobile phase, consisting of water and acetonitrile in a ratio of 70:30 (V:V), was loaded onto an ACQUITY UPLC BEH Shield RP18 column (1.7 μm, 2.1 mm ×100 mm, Waters) at a flow rate of 0.3 mL/min. The liposomes were diluted and disrupted with 50-fold methanol to obtain total amount of TPL in the liposomes. The equation for calculating entrapment efficiency (EE%) of TPL is as following:

$$EE\left(\%\right)=\frac{W_{total\;drug}-W_{unencapsulated\;drug}}{W_{total\;drug}}\times 100\%$$

Where *W_total drug_* and *W_unencapsulated drug_* are the total amount of TPL and the unencapsulated TPL, respectively.

### *In vitro* drug release study

Briefly, 1 mL of dl-TPL-lip, CA IX-TPL-lip, CPP33-TPL-lip, TPL-lip, and free TPL solution (0.05 mg/mL) was placed into dialysis tubes with a molecular cut-off of MWCO 12–14 kDa and dialyzed in 10 mL of PBS (pH 7.4) at 37 °C. At predetermined time intervals, 100 μL of release medium was taken out and replaced with equal volume of fresh medium. The concentration of TPL in release medium was then determined by UPLC.

### Wound healing assay

For monolayer cells study of dl-TPL-lip, A549 cells were exposed to hypoxia (in a modular incubator chamber purged with 1% O_2_, 5% CO_2_, and balance N_2_) at 37 °C to induce the CA IX expression (CA IX-positive cells).

For wound healing assay, 5 × 10^5^ of A549 cells were seeded and cultured in 12-well plates maintained under hypoxia until 90% confluence (Chereddy et al., 2014). The confluent cells were wounded with a 10-μL pipette tip to get two cell islands (Liang et al., 2015). The cells were washed twice with PBS to remove debris, then incubated with blank medium (as control), free TPL, TPL-lip, CA IX-TPL-lip, CPP33-TPL-lip, dl-TPL-lip at a concentration of 100 μM TPL for 4 h, respectively. After 4 h treatment, the cells were washed with PBS and then incubated with fresh medium at 37 °C for additional 68 h. Photographs of the *in vitro* wounds were taken at the beginning (0 h) and 72 h with a microscope (Leica DMI3000 B, Wetzlar and Mannheim, Germany).

### *In vitro* apoptosis assay

Apoptosis of A549 cells was determined using an Annexin V-FITC Apoptosis Detection Kit I (BD Pharmingen^TM^, Franklin Lakes, NJ, USA) (Liang et al., 2015). Briefly, 5 × 10^5^ of A549 cells/well were seeded into 6-well plates and allowed to grow 24 h under hypoxia. Free TPL, TPL-lip, CA IX-TPL-lip, CPP33-TPL-lip, dl-TPL-lip were added to each well with 100 μM of TPL and incubated for 4 h. Cells alone were incubated with fresh culture medium as control. The cells were washed with PBS to remove the unbound drugs and then incubated with fresh medium at 37 °C for an additional 20 h. Cells were collected and washed with cold PBS twice followed by suspension in 500 μL binding buffer. Then, 5 μL of Annexin V-FITC and 5 μL of PI were added to the cell suspensions. After 15 min incubation in the dark, the double-stained cells were immediately analyzed by FACScan flow cytometry (Becton Dickinson, San Jose, CA).

### Penetration in 3 D tumor spheroids

To prepare 3 D tumor spheroids, A549 cells (1 × 10^3^ cells/well) were seeded in 2% (*w/v*) low-melting point agarose-coated 96-well plates (Liu et al., 2014). After seven days, the uniform and compacted spheroids were selected for the following study.

For uptake study, the 3 D tumor spheroids were transferred to a confocal dish and incubated with NBD-DPPE-labeled CPP33-lip, CA IX-lip, dl-lip and lip, respectively. After incubation for 4 h at 37 °C, the spheroids were rinsed twice with cold PBS and were fixed with 4% paraformaldehyde for 15 min. Z-stack images of spheroids from the top to 100 μm were collected using confocal laser scanning microscope (CLSM).

### Cytotoxic damage assay in 3 D tumor spheroids

Cell viability of the spheroids was determined after four days’ treatment of seven-day-old spheroids. Free TPL, TPL-lip, CA IX-TPL-lip, CPP33-TPL-lip or dl-TPL-lip was added to each well with 100 μM of TPL and incubated for four days. The morphology of the spheroids was observed using a microscope (Leica DMI3000 B, Germany) before MTT assay. Then, MTT solution (20 μL, 5 mg/mL in PBS) was added to each well followed by gentle mixing to disrupt the spheroids. After 4 h incubation, the medium was removed and 100 μL/well of DMSO was added to dissolve the formazan by shaking for 15 min. The absorbance was measured with a microplate reader (Bio-rad Laboratories, Berkeley, USA) at a wavelength of 570 nm.

## Pharmacokinetics study of dl-TPL-lip after pulmonary administration to rats

Sprague–Dawley (SD) rats were divided randomly into five groups (*n* = four per group). Rats were fasted for 12 h before the experiment, but allowed water *ad libitum*. Free TPL, TPL-lip, CPP33-TPL-lip, CA IX-TPL-lip, and dl-TPL-lip were administered directly via pulmonary delivery using a Microsprayer Aerosolizer Pulmonary Aerosol Kit for Mouse Model PAK-MSA (Penn-Century, Inc. Wyndmoor, PA19038, USA) at a dose of 0.5 mg/kg. Blood samples (0.4 mL) were obtained before dosing and at designated time points (0.083, 0.5, 1, 2, 4, 6, 8, 12 h). The samples were collected into heparinized tube from the suborbital veins and were centrifuged for 10 min at 4,000 rpm. The plasma was analyzed by Agilent 1290 Ultra High Performance Liquid Chromatograph with Agilent 6460 Triple Quadrupole Mass Spectrometer system (UHPLC-QQQ MS/MS, Santa Clara, USA) (Xue et al., 2012; Zhuang et al., 2013).

### Establishment of orthotopic lung tumor models

An orthotopic lung tumor-bearing mice model was established as previously reported. Briefly, the male BALB/c nu/nu mice (7–8 weeks) were anesthetized with 5% of chloral hydrate and 100 μL of A549-Red-FLuc cells (2 × 10^6^ cells) solution in Matrigel (1:1). Experimental solutions were injected percutaneously into the right lateral thorax of each mouse using a 1 mL syringe with 29-gauge needle. On day seven after tumor cell inoculation, bioluminescence was measured using the IVIS Lumina XR system (Caliper, Hopking, MA) to check the successful establishment of lung cancer. d-luciferin substrate (i.p., 150 mg/kg in 0.9% saline solution) was injected 15 min before bioluminescence imaging. On day 19 after tumor cell inoculation, the mice were sacrificed and lungs were immediately resected.

### Anti-cancer effect of dl-TPL-lip in orthotopic mice model of lung cancer

On day seven following tumor cells inoculation, the orthothopic lung tumor-bearing mice (*n* = 30) were grouped into six treatment groups based on equivalent signal intensity of the tumor bioluminescence: Free TPL, TPL-lip, CA IX-TPL-lip, CPP33-TPL-lip, dl-TPL-lip, and control group with saline. Five treatment groups received their respective TPL formulation every three days four times via pulmonary delivery at 0.3 mg/kg. Tumor growth was monitored weekly by bioluminescence imaging as before, during, and after the TPL treatment. Body weight of the mice was recorded weekly during the course of the study. At the conclusion of the study, mice were sacrificed, and lungs were immediately resected and photographed.

### Data analysis

The TPL pharmacokinetic parameters in mice after endotracheal administration were calculated and summarized by DAS 2.1.1. Statistical analyses for anti-cancer effect study were evaluated by two-way ANOVA with Bonferroni posttests using GraphPad Prism 6 (GraphPad Software, Inc, La Jolla, USA).

## Results and discussions

### Synthesis of DSPE-PEG-MAL-CPP33 conjugate

**Figure 1.**
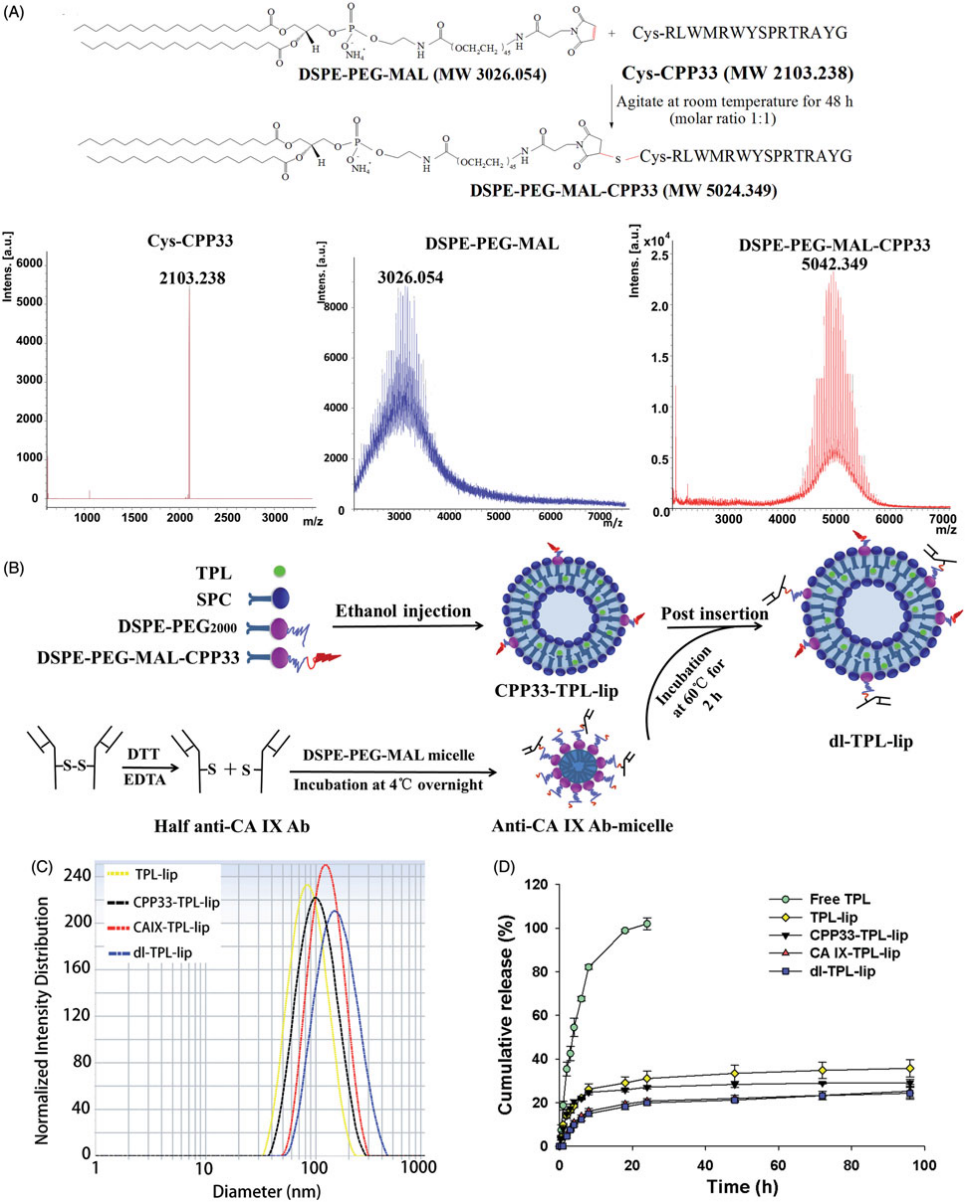
(A) Synthesis of DSPE-PEG-MAL-CPP33 conjugate, MALDI-TOF-MS spectra of CPP33, DSPE-PEGMAL, and DSPE-PEG-MAL-CPP33 conjugate; (B) Illustration of the preparation of dl-TPL-lip; (C) Size distribution of different TPL liposomal formulations; (D) TPL release profiles of free TPL, TPL-lip, CPP33-TPLlip, CA IX-TPL-lip, and dl-TPL-lip in PBS (pH 7.4) over 96 h (*n* = 3, mean ± SD).

DSPE-PEG-MAL-CPP33 conjugate was synthesized by coupling the thiol group of Cys-CPP33 with the maleimide group of DSPE-PEG_2000_-MAL (Yang et al., 2014). The synthesis of the DSPE-PEG-MAL-CPP33 conjugate is illustrated in Figure 1(A). As shown, DSPE-PEG-MAL-CPP33 was synthesized via the thiol-ene “click” reaction of the maleimide group with the sulfhydryl group of CPP33. MALDI-TOF MS showed that the major peak had a 5042.349 mass-charge ratio; this matched our theoretical calculations and therefore confirmed success of the synthesis.

### Preparation and characterization of dl-TPL-lip

Dl-TPL-lip was fabricated via ethanol injection followed by the post-insertion (Figure 1(B)). As listed in Table 1, the mean diameters of all TPL liposomal formulations ranged from 87.4 to 137.6 nm, and the PDI was in a range of 0.07–0.17. The increased diameters suggested that the anchors are presented on the surface of liposomes. The EE% was in a range of 86.3%–92.3%, suggesting that TPL was well-loaded within liposomes. The representative particle size distributions of TPL liposomal formulations are shown in Figure 1(C).

**Table 1.** Characteristics of TPL liposomal formulations (*n* = 3, mean ± SD).

Formulation	Mean diameter (nm)	Polydispersity	Encapsulation efficiency (%)
TPL-lip	87.4 ± 1.6	0.066 ± 0.019	87.7 ± 2.4
CPP33-TPL-lip	96.4 ± 0.7	0.111 ± 0.024	86.7 ± 1.2
CA IX-TPL-lip	113.1 ± 4.5	0.160 ± 0.017	92.3 ± 2.7
dl-TPL-lip	137.6 ± 0.8	0.173 ± 0.010	86.3 ± 1.9

### *In vitro* drug release study

For the *in vitro* release study, a dialysis method was used under sink conditions. Compared with the rapid release profile of free TPL, the *in vitro* release rate of TPL from TPL-lip, CPP33-TPL-lip, CA IX-TPL-lip, and dl-TPL-lip was evenly sustained release without burst release (Figure 1(D)). The similarity in release profiles of the four liposomal formulations demonstrates that modification with CPP33 and/or anti-CA IX antibody do not remarkably influence the release kinetics of the liposomes. The slight initial differences between liposome formulations and release behavior may distribute to the presence of the ligands, which hold up the release of free TPL. In addition, this relatively stable release of TPL from the liposomes may prevent a rapid leakage during delivery and ensure efficient accumulation of TPL at tumor sites, both characteristics are beneficial for tumor therapy (Cai et al., 2014a).

### Wound healing study

**Figure 2.**
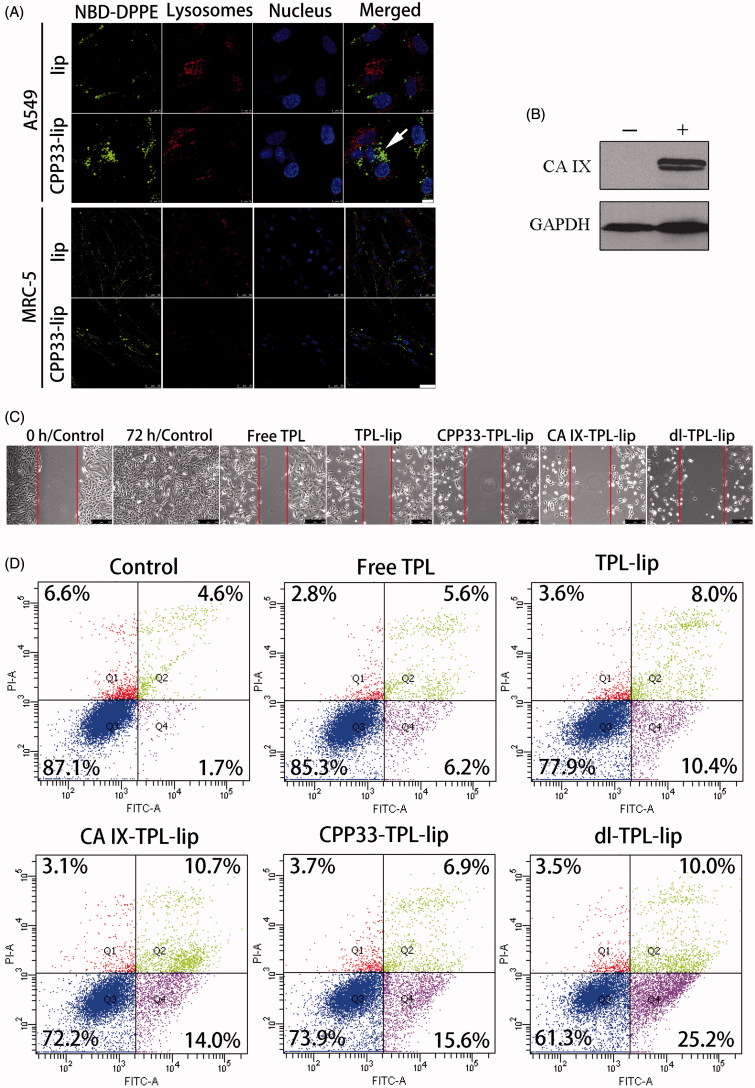
(A) Cellular uptake of NBD-DPPE-labeled lip and CPP33- lip by A549 cells (scale bars, 10 μm) and normal lung fibroblast MRC-5 cells (scale bars, 50 μm) by CLSM; (B) Detection of CA IX expression in A549 cells after hypoxic (+) and normoxic (−) treatment for 20 h; GAPDH was used as the loading control; (C) Wound healing study (scratch assay) on CA IX-positive A549 cells observed with microscope (scale bar is 250 μm); (BD) Apoptosis assay on CA IX-positive A549 cells by flow cytometry after treatment with Free TPL, TPL-lip, CPP33-TPL-lip, CA IX-TPL-lip and dl-TPL-lip (TPL concentration 100 μM).

Although CPP33 has been reported as a tumor lineage-homing cell-penetrating peptide that can selectively penetrate lung cancer cells, whether it would retain these properties after inclusion on liposomes was unknown. Herein, the ability of CPP33-modified liposomes to selectively penetrate A549 cells rather than normal cells was at first verified (Figure 2(A)); success in this test provided a solid foundation for the following study on dl-TPL-lip. To mimic the hypoxic microenvironment of a solid tumor, lung cancer cells were incubated under a hypoxic condition (1% O_2_, 5% CO_2_, and 94% N_2_) and the expression of CA IX in lung cancer cells (CA IX-positive cells) was confirmed by Western blot (Figure 2(B)) (Wong et al., 2014).

The wound healing study was carried out to investigate the ability of dl-TPL-lip to inhibit cell motility and metastasis. After a wound had been artificially created by scratching a cell monolayer, A549 cells were treated with different TPL formulations for 4 h and then allowed to repair for additional 68 h at 37 °C. As shown in Figure 2(C), the CA IX-positive A549 cells showed efficient proliferation and migration into the damaged area in the control. In contrast, all the TPL formulations greatly depressed both proliferation and migratory activities of CA IX-positive A549 cells. The dl-TPL-lip resulted in the greatest inhibitory effects in CA IX-positive A549 cells as compared to all other treatment. Dual-ligand modification enhanced the binding of liposomes to the tumor cells and enhanced penetration of TPL into tumor cells.

### Apoptosis assay

To verify the wound healing experimental results, we investigated the level of apoptosis in cells after treatment with TPL formulations. Annexin V is a Ca^2+^-dependent phospholipid-binding protein with high affinity for phosphatidylserine (PS). In the early stages of apoptosis, the PS of cell membrane phospholipid is translocated from the inner to the outer leaflet of the plasma membrane where it can more readily bind with Annexin V. PI was used as a vital dye to distinguish cells with damaged membrane from intact cells. In late apoptotic cells, PI can penetrate into the cells and stain the nuclei. Figure 2(D) shows representative dot plots of the double-stained cell populations. Dl-TPL-lip exhibited the strongest effect on inducing apoptosis in CA IX-positive A549 cells compared to single ligand-modified liposomes with anti-CA IX antibody or CPP33. This result indicates that the simultaneous modification of liposomes with CPP33 and anti-CA IX antibody enhanced the apoptotic effect of TPL.

### Penetration in 3 D tumor spheroids

**Figure 3.**
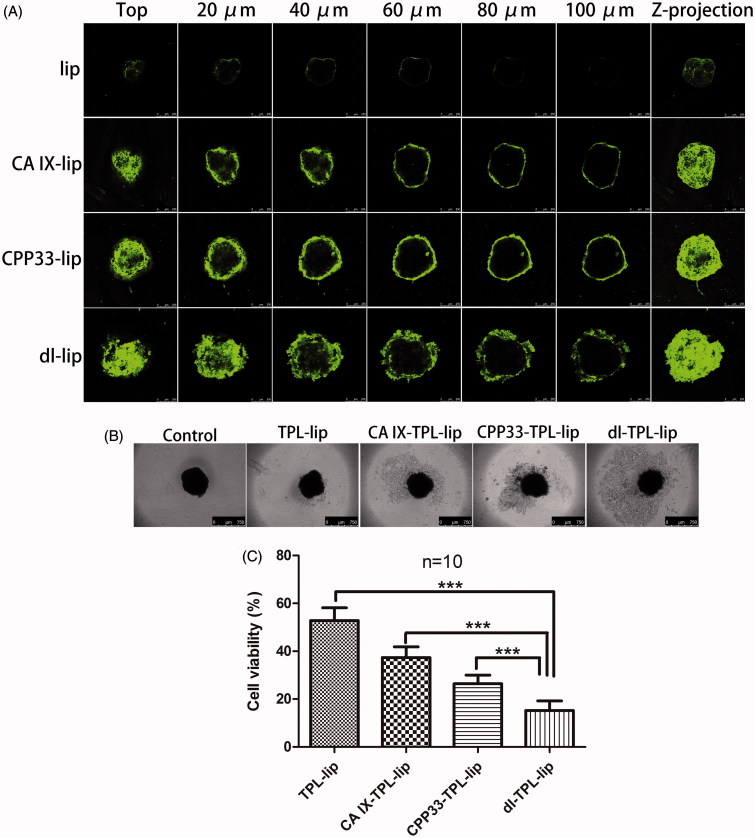
(A) Penetration of NBD-DPPE-labeled lip, CA IX-lip, CPP33-lip, and dl-lip throughout A549 3 D tumor spheroids by confocal laser scanning microscope using Z-stacking imaging with 20 μm intervals (scale bars, 250 μm). (B) Representative brightfield images and (C) Cell viability of spheroids after treated with TPL-lip, CA IX-TPL-lip, CPP33-TPL-lip and dl-TPL-lip for 4 days with TPL concentration of 100 μM (mean ± SD, *n* = 10). Scale bars, 750 μm; ***, *p* < .001.

*In vitro* 3 D tumor spheroids are currently the most effective models to qualitatively and quantitatively assess drug penetration. Compared to a traditional monolayer culture system, the 3 D tumor spheroids are more closely similar to those *in vivo* (Mehta et al., 2012). 3 D tumor spheroids display many of the characteristics of natural tissue including a core region of hypoxia, necrotic cells, and the production of an extracellular matrix; these characteristics have been demonstrated to block the penetration of traditional drugs and nanoparticles into solid tumors. Therefore, 3 D tumor spheroids models were used in this study to evaluate the penetration ability of dl-lip. Firstly, the expression of CA IX on the spheroids was confirmed in our previous study. (Lin et al., 2017) Then, the penetration of dl-lip was further studied here. Clearly, dl-lip penetrated more deeply into different layer of the spheroids as compared to non-modified liposomes, CA IX-lip, and CPP33-lip (Figure 3(A)). This result suggests that dual-ligand modification can enhance the penetration of the liposomes.

### Cytotoxic damage assay in 3 D tumor spheroids

To further evaluate the cytotoxic damage of TPL liposomal formulations in 3 D tumor spheroids, spheroids with homogeneous volume and shape were exposed to TPL-lip, CA IX-TPL-lip, CPP33-TPL-lip, and dl-TPL-lip for four days. As shown in Figure 3(B), a well-defined spheroid rim and compact structure was observed in the control spheroids, while a smeared rim was observed in all liposomal treatment groups. In contrast, an evident collapse of spheroid architecture with more shedding was seen in dl-TPL-lip. This collapse might be explained by the enhanced binding, penetration, and consequent cytotoxicity of TPL to the peripheral cell layers. In agreement with the results from bright-field microscope analysis, MTT assay showed that dl-TPL-lip induced significantly higher cytotoxicity compared with other TPL liposomal formulations (Figure 3(C)), with low variability of 15.24%. This result reveals that dual-ligand modification with anti-CA IX antibody and CPP33 on the surface of liposomes has great potential for use in lung cancer therapy.

### Pharmacokinetics study of dl-TPL-lip after pulmonary administration to rats

**Figure 4.**
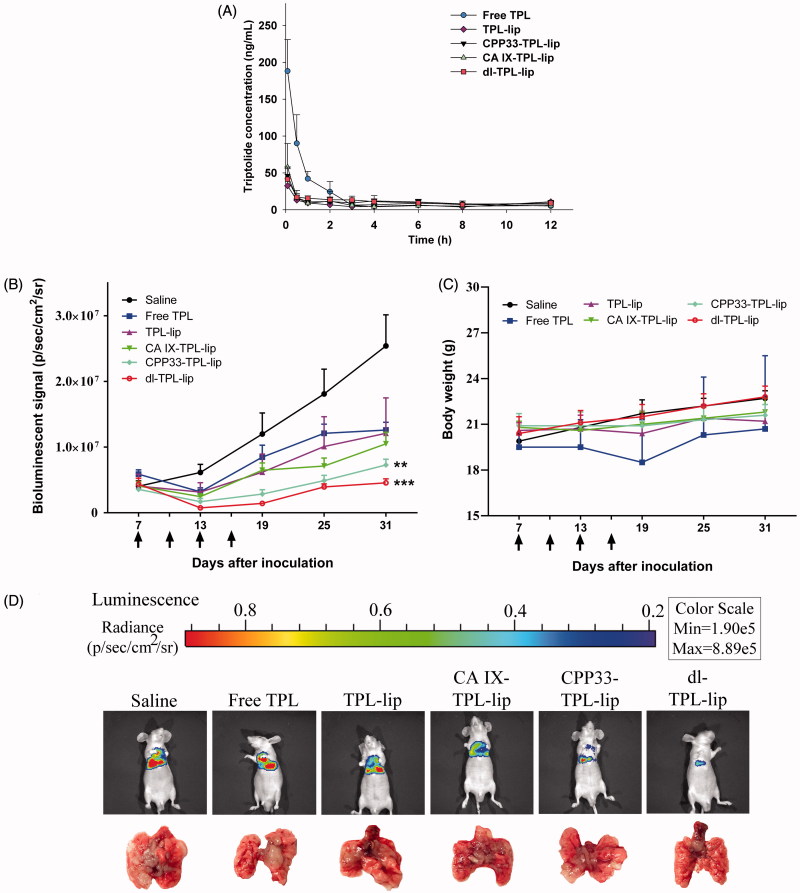
(A) Plasma concentrations of TPL in normal Sprague-Dawley rats that received endotracheal administration of different TPL formulations at a TPL dose of 0.5 mg/kg (*n* = 4, mean ± SD). (B) Changes in tumor burden monitored by the IVIS Lumina XR system (*n* = 5, mean ± SEM); Arrows indicate time of administration (on day 7, 13, 19, 25); **p < .01, ***p < .001, compared with saline. (C) Changes in body weight of orthotopic lung tumor bearing mice. (D) Representative bioluminescent images and corresponding lung tissue of the mice at day 31 after tumor inoculation.

For pharmacokinetics study, a UHPLC-QQQ MS/MS analytical method was established and validated (Supplementary Figures S1 and S2). The pharmacokinetic profiles of TPL from TPL solution and liposomal formulations were compared by determining the concentration of TPL in rat plasma after a single endotracheal administration at 0.5 mg/kg. The main pharmacokinetic parameters were analyzed by DAS 2.1.1. As shown in Figure 4(A) and Table 2, a three-fold higher maximum concentration (*C*max) of TPL was detected in the blood of rats administered with free TPL compared to liposomal TPL. No significant differences were shown in the liposomal formulations. Since our previous bio-distribution study of CA IX-Lips via endotracheal administration showed specific localization of the liposomes in lung (Lin et al., 2017), we hypothesized that this change in pharmacokinetic behaviors resulted from the ability of the liposomal formulations to retain the encapsulated TPL in the lung after endotracheal administration (Gill et al., 2011; Chen et al., 2012b; Luo et al., 2016), such that it did not circulate in the whole body. This behavior is ideal for lung cancer therapy. The low *C*max in liposomal TPL implies low distribution of TPL in systemic circulation; such behavior is favorable for reducing unwanted side effects. In contrast, free TPL rapidly enters systemic circulation, which means its concentration in the lung is reduced and it is freely available to other organs.

**Table 2.** The pharmacokinetic parameters of different TPL formulations after endotracheal administration (0.5 mg/kg) in mice (*n* = 4).

Parameters	Unit	Free TPL	TPL-lip	CA IX-TPL-lip	CPP33-TPL-lip	dl-TPL-lip
AUC(0–*t*)	μg/L*h	185.89 ± 36.48	84.28 ± 10.60	126.02 ± 12.20	68.33 ± 27.41	131.54 ± 10.46
AUC(0–∞)	μg/L*h	224.87 ± 63.76	228.74 ± 67.72	191.83 ± 89.75	125.01 ± 76.18	244.54 ± 63.02
*t*1/2	H	2.98 ± 0.54	9.74 ± 5.68	6.39 ± 3.97	7.68 ± 9.68	9.96 ± 6.63
*T*max	h	0.083	0.083	0.083	0.083	0.083
*C*max	μg/L	188.17 ± 42.71	32.56 ± 9.37	46.25 ± 11.96	57.71 ± 32.15	41.55 ± 16.42

### Establishment of orthotopic lung tumor models

In this study, we used a firefly luciferase gene-transduced cell line (A549-Red-Fluc) for intrathoracic injection to create an orthotopic lung tumor-bearing model in nude mice, and we quantitatively monitored tumor growth. To confirm that the radiance of cells could be used to characterize tumor growth, the relationship of cell number with radiance was at first determined *in vitro* before inoculating the mice. As shown in Figure S3, the average radiance increased with cell number in a linear relationship (R^2^ = 0.9955), which warranted its application *in vivo*. As illustrated, after tumor cell inoculation, the establishment of tumors was confirmed using an IVIS imaging system and checked by dissection. Results confirmed that a solid tumor was induced in the lung and showed no metastasis to other organs.

### Anti-cancer effect of dl-TPL-lip in orthotopic mice model of lung cancer

The anti-cancer effect of dl-TPL-lip was further evaluated in orthotopic lung tumor-bearing mice via pulmonary delivery at a dose of 0.3 mg/kg; each mouse was given four doses – one dose every three days four times (Supplementary Figure S4). The tumor progress and body weights of mice were monitored. On day 31, the tumors of control group had increased ∼6-fold, while those of the free TPL, TPL-lip, and CA IX-TPL-lip had increased ∼3-fold, CPP33-TPL-lip had increased ∼2-fold, and dl-TPL-lip had increased only ∼1-fold (Figure 4(B)). No significant difference in body weight change was observed between different treatment groups and the control, indicating no obvious systemic toxicity of TPL formulations (Figure 4(C)). Figure 4(D) shows representative bioluminescence images and photographs of the dissected lungs of each group on the last day of the study. The dl-TPL-lip exhibited the highest anti-cancer effect compared to non-modified liposomes and single ligand-modified liposomes. This result indicates that dual-ligand modification with anti-CA IX Ab and CPP33 on liposomes offers distinct advantages over single modification. The dual-ligand modification appears to enhance the anti-cancer effect of TPL by simultaneously increasing targeting and internalization of TPL-loaded liposomes.

## Conclusions

In summary, anti-CA IX antibody and CPP33 dual-ligand TPL-loaded liposomes with uniform size (137.6 ± 0.8) and high-encapsulation efficiency (86.3 ± 1.9%) were successfully developed in this study. The dl-TPL-lip showed a sustained release in pH 7.4 PBS. The dl-TPL-lip could inhibit cellular proliferation and migration into the damaged area and enhance cellular apoptosis. The penetration in 3 D tumor spheroids showed that dl-lip has much better penetrating ability than either non-modified liposomes or single-ligand-modified liposomes. Moreover, the dl-TPL-lip showed a stronger inhibitory effect on 3 D tumor spheroids. Dl-TPL-lip and other TPL liposomal formulations all exhibited a lower drug concentration in plasma compared with TPL solution, which suggested lower drug concentration in systemic circulation, hence lower exposure to other organs. This result demonstrated that encapsulation of TPL in liposomes alters the distribution behavior of TPL. In the anti-cancer effect study, dl-TPL-lip invoked the strongest anti-cancer response without obvious systemic toxicity. This suggests that the simultaneous modification of TPL-loaded liposomes with anti-CA IX antibody and CPP33 increases the liposome’s therapeutic effects. The study provides a potentially very promising dual-ligand modified liposomal vehicle for local and targeted delivery of anti-cancer drugs.

## Supplementary Material

IDRD_Yang_et_al_Supplemental_Content.pdf
