# Supplementary material for: Dual-ligand modified liposomes provide effective local targeted delivery of lung-cancer drug by antibody and tumor lineage-homing cell-penetrating peptide
Source: Drug Deliv. 2018 Jan 15;25(1):256–66. doi: 10.1080/10717544.2018.1425777 (PMC6058720; doi:10.1080/10717544.2018.1425777)
Supplement: IDRD_Yang_et_al_Supplemental_Content.pdf [file IDRD_A_1425777_SM8742.pdf]

Supplementary information

Dual-ligand modified liposomes provide effective local targeted delivery of lung-cancer drug by antibody and tumor lineage-homing cell-penetrating peptide

Congcong Lin[1], Xue Zhang[1], Hubiao Chen[1], Zhaoxiang Bian[1], Ge Zhang[1], Muhammad Kashif Riaz[1], Deependra Tyagi[1], Ge Lin[3], Yanbo Zhang[4], Jinjin Wang[2], Aiping Lu[1,2*], Zhijun Yang[1,2 *]

[1]School of Chinese Medicine, Hong Kong Baptist University, 7 Baptist University Road, Kowloon Tong, Hong Kong, China

[2]Changshu Research Institute, Hong Kong Baptist University, Changshu Economic and Technological Development (CETD) Zone, Changshu 215500, China

[3]School of Biomedical Sciences, Chinese University of Hong Kong, Area 39, CUHK, Shatin, NT, Hong Kong, China

[4]School of Chinese Medicine, Li Ka Shing Faculty of Medicine, The University of Hong Kong, 10 Sassoon Road, Pokfulam, Hong Kong, China

[*]Corresponding author.

Correspondence and requests for materials should be addressed to Z.Y. (email: yzhijun@hkbu.edu.hk; Tel.: +852-3411-2961; fax: +852-34112461)

Cellular uptake study

A549 cells, a human non-small cell lung cancer cell line and MRC-5, a human lung fibroblast cell line were seeded to confocal dish and allowed to incubate for 24 h at a density of 1×10^5 cells per well. Then, cells were incubated with NBD-DPPE labeled CPP33-lip and lip (final NBD-DPPE concentration 4 μg/mL) for 4 h at 37 ℃. 30 min before the end of the treatment, LysoTracker Red (50 nM) was added to stain the lysosomes. The cells were washed three times with PBS, followed by fixation with 4% paraformaldehyde for 15 min. The nuclei were stained with Hoechst 33342 (2.5 μg/mL) for 15 min at 37 ℃. Finally, the cells were washed another three times with PBS and visualized by confocal laser scanning microscope (CLSM).

Detection of CA IX expression in cell cultures

A549 cells were exposed to normoxia (humidified air with 5% CO_2) or hypoxia (in a Modular Incubator Chamber purged with 1% O_2, 5% CO_2 and 94% N_2) at 37 ℃ for 20 h, followed by CA IX detection by Western blot. The samples were denatured with loading buffer and 20 μL of each sample was loaded into 8% SDS-PAGE gels under reducing conditions, then electrotransferred to a polyvinylidene difluoride (PVDF) membranes. The blots were blocked with 5% nonfat milk for 1 h, followed by incubation with anti-CA IX primary antibody overnight at 4 ℃. Then the membrane was incubated with the goat anti-rabbit HRP-conjugated secondary antibody at room temperature for 1 h. A SuperSignal West Pico Chemiluminescent Substrate (Thermo Fisher Scientific) was used for HRP-based detection of the anti-CA IX antibody.

Analytical method for pharmacokinetics study

Hydrocortisone was added (50 μL, 200 ng/mL) as an internal standard (IS) to 0.2 mL of plasma. The samples were mixed, and extracted with ethyl acetate (750 μL) followed by vortexing for 4min and centrifuging for 10min at 8000 rpm. The upper organic phase was carefully transferred and evaporated to dryness in vacuum. The residues were dissolved in 100μL methanol and centrifuged at 4000rpm for 10 min, then the supernatant was collected and injected into the Agilent 1290 Ultra High

Performance Liquid Chromatograph with Agilent 6460 Triple Quadrupole Mass Spectrometer system (UHPLC-QQQ MS/MS).

Chromatography conditions: Waters Acquity BEH C18 (2.1 ×50 mm); Mobile phase 0.1% formic acid in water: 0.1% formic acid in acetonitrile =80:20 v/v; Flow rate 0.35 mL/min; Running time 10 min.

Mass spectrometer conditions: Ion Source Agilent Jet Stream electro spray ionization (AJS ESI); Positive mode; Gas Temperature 300 ℃; Gas flow rate 8 L/min; Nebulizer 40 psi; SheathGasHeater 350 ℃; SheathGasFlow 8 L/min; Capillary 3500 V; VCharging 1000 V.

Firstly, optimization of mass spectrometry was initiated by automatic tuning of the instrument (Figure S1). Method validation was conducted before the sample analysis. The specificity of the established method was assessed by comparing the chromatograms of blank plasma, blank plasma with TPL, blank plasma with IS, and plasma after endotracheal administration (Figure S2). The calibration curve in rat plasma was linear over the ranges 10-2500 ng/mL in rat plasma (y=0.375035x + 0.025498, R^2=0.9946). The extraction recoveries were higher than 90%, while accuracy and precision both intra-day and inter-day were less than 10%. The method was sufficiently accurate and consistent for proceeding for the biological analysis of TPL (Table S1). Therefore, the established method was applied to the analysis of rat plasma samples.

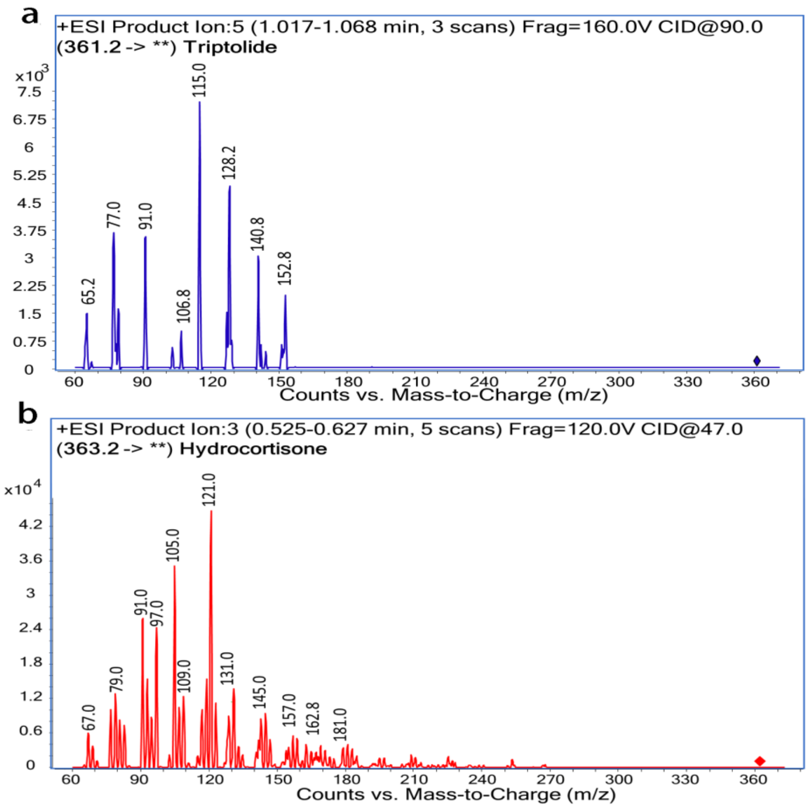

Fig. S1. Mass spectra of (a) TPL and (b) IS (Hydrocortisone).

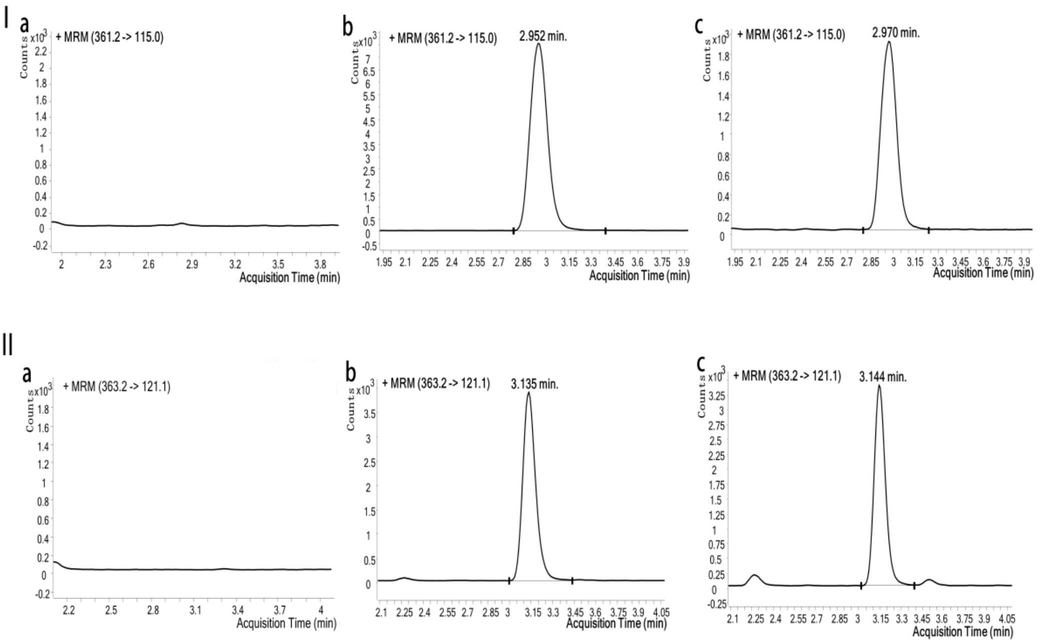

Fig. S2. Representative MRM chromatograms of TPL (Ⅰ) and IS (Ⅱ). (a) blank plasma, (b) blank plasma spiked with TPL and IS, (c) plasma sample after endotracheal adiminstration of 5 min with a dose of 0.5 mg/kg.

Table S1. Extraction recovery, accuracy and precision data of TPL in plasma (n=5).

Concentration (ng/mL)	Extraction recovery% (mean±SD)	Accuracy (RE%)		Precision (RSD%)	
		Intra-day	Inter-day	Intra-day	Inter-day
20	96.07±5.61	0.73	0.25	9.55	5.61
313	95.16±6.52	0.58	0.47	8.33	7.82
2500	94.72±3.17	0.70	0.55	9.90	8.33

Relationship of cell number with radiance

Luciferase gene transduced cell line (A549-Red-Fluc) was used to create an orthotopic lung tumor bearing model by intrathoracic injection. The relationship of cell number with radiance was firstly determined *in vitro* before inoculating into the mice. A549-Red-Fluc cells were cultured in Dulbecco's modified Eagle's medium (DMEM) with GlutaMAX supplemented with 10% fetal bovine serum, 100 U/mL penicillin, and 100 μg/mL streptomycin. The cells were seeded at a density of 1×10^6 into a T75 flask, and grown for 2 days. Thereafter, the cells were trypsinized and collected. Cells were pelleted by centrifuging at 1000 rpm for 5 min; the old medium was removed. Cells were washed and resuspended with PBS. 100 μL of various numbers of cells (0, 1.562, 3.125, 6.25, 12.5, 25, 50, 100 $\times10^4$) were transferred into black 96-well plates. For luciferase expression, 100 μL of D-luciferin was added into each well. The luminescence signal was determined by the IVIS Lumina XR system (Caliper Life Sciences) （Figure S3）.

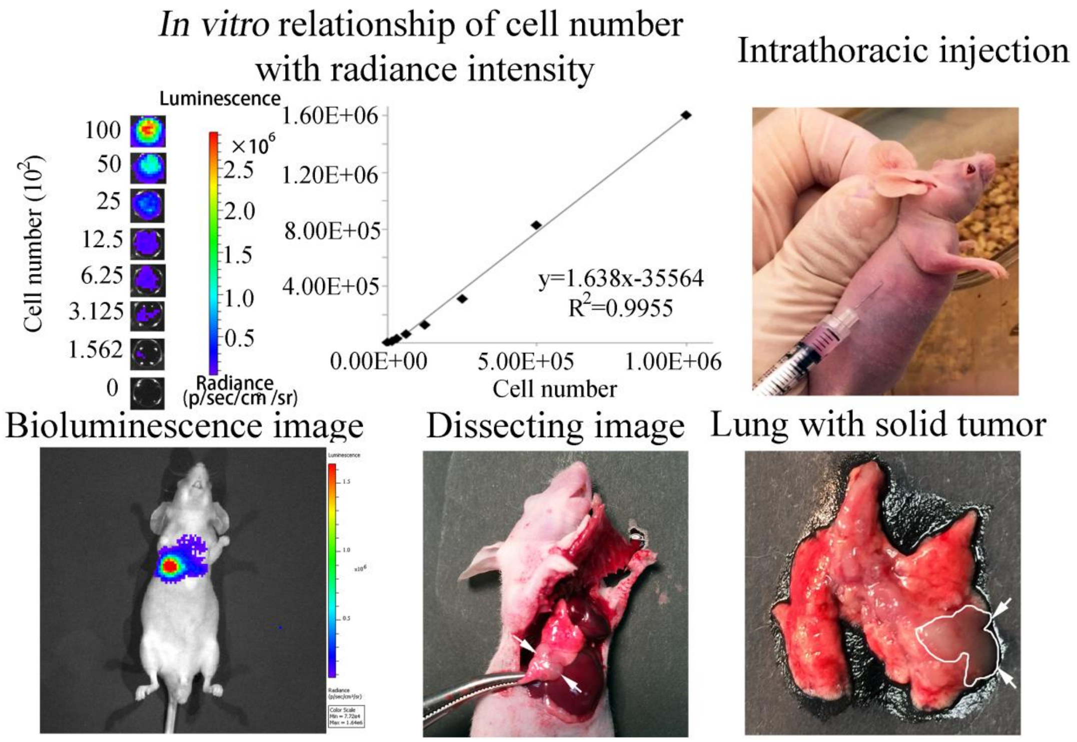

Fig. S3. Establishment of orthotopic lung tumor mode.

Pulmonary administration of the drug

For endotracheal administration, the mouse was firstly anesthetized with 5% of chloral hydrate at a dose of 300 mg/kg by intraperitoneal injection. Then the mice were placed in a neck vertical position. The tongue of mouse was gently pulled out with tweezes. A small transparent tube was inserted under the tongue to the trachea of mouse. The treatment formulations were sprayed to the trachea using Microsprayer® Aerosolizer Pulmonary Aerosol kit for Mouse Model PAK-MSA (Penn-Century, Inc. Wyndmoor, PA19038 USA) （Figure S4）.

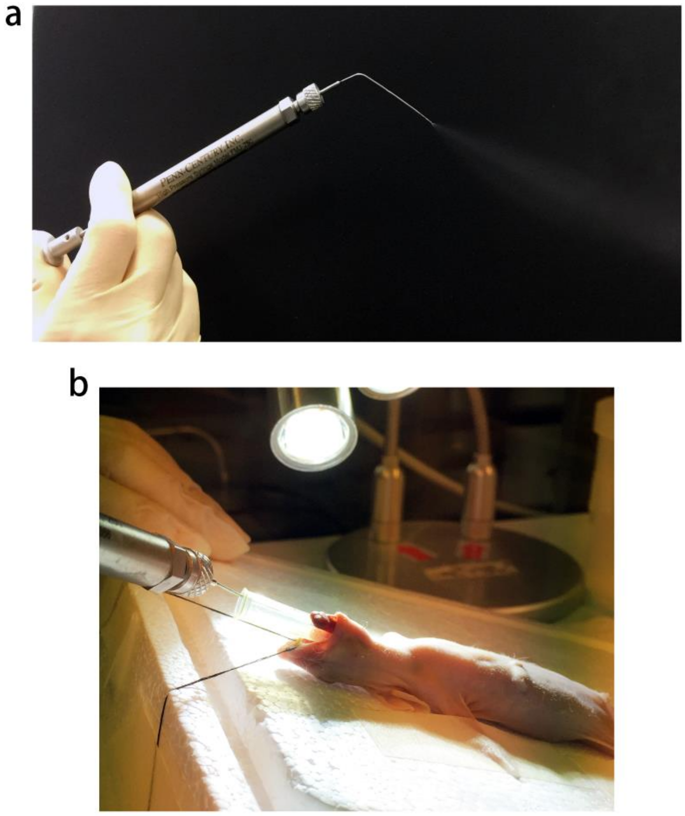

Fig. S4. Illustration for endotracheal administration. (a) Microsprayer® Aerosolizer Pulmonary Aerosol kit for Mouse Model PAK-MSA; (b) Endotracheal administration to the nude mouse.
